# RNA-sequencing revisited data shed new light on wooden breast myopathy

**DOI:** 10.1016/j.psj.2024.103902

**Published:** 2024-05-25

**Authors:** Martina Bordini, Ziqing Wang, Francesca Soglia, Massimiliano Petracci, Carl J. Schmidt, Behnam Abasht

**Affiliations:** ⁎ Department of Agricultural and Food Sciences, Alma Mater Studiorum – University of Bologna, Cesena, Italy; † Department of Animal and Food Sciences, University of Delaware, Newark, DE, USA

**Keywords:** wooden breast, differential expression analysis, mitochondrial dysfunction, energy metabolism, muscular hypertrophy

## Abstract

Wooden Breast (**WB**) abnormality represents one of the major challenges that the poultry industry has faced in the last 10 years. Despite the enormous progress in understanding the mechanisms underlying WB, the precise initial causes remain to be clarified. In this scenario, the present research is intended to characterize the gene expression profiles of broiler *Pectoralis major* muscles affected by WB, comparing them to the unaffected counterpart, to provide new insights into the biological mechanisms underlying this defect and potentially identifying novel genes likely involved in its occurrence. To this purpose, data obtained in a previous study through the RNA-sequencing technology have been used to identify differentially expressed genes (**DEGs**) between 6 affected and 5 unaffected broilers’ breast muscles, by using the newest reference genome assembly for *Gallus gallus* (**GRCg7b**). Also, to deeply investigate molecular and biological pathways involved in the WB progression, pathways analyses have been performed. The results achieved through the differential gene expression analysis mainly evidenced the downregulation of glycogen metabolic processes, gluconeogenesis, and tricarboxylic acid cycle in WB muscles, thus corroborating the evidence of a dysregulated energy metabolism characterizing breasts affected by this abnormality. Also, genes related to hypertrophic muscle growth have been identified as differentially expressed (e.g., *WFIKKN1*). Together with that, a downregulation of genes involved in mitochondrial biogenesis and functionality has been detected. Among them, *PPARGC1A* and *PPARGC1B* chicken genes are particularly noteworthy. These genes not only have essential roles in regulating mitochondrial biogenesis but also play pivotal roles in maintaining glucose and energy homeostasis. In view of that, their downregulation in WB-affected muscle may be considered as potentially related to both the mitochondrial dysfunction and altered glucose metabolism in WB muscles, and their key involvement in the molecular alterations characterizing this muscular abnormality might be hypothesized.

## INTRODUCTION

Wooden Breast (**WB**) abnormality represents one of the major challenges that the poultry industry has faced over the past 10 years. Indeed, great effort has been put into improving the productive performance of modern broilers, for example, growth rate, breast meat yield, and feed efficiency (Abasht et al., 2019b; Bottje et al., 2021; Soglia et al., 2021; Alnahhas et al., 2023). However, these improvements have indirectly contributed to the development of several growth-related muscular defects mainly affecting the *Pectoralis major* muscle of fast-growing genotypes (Petracci et al., 2019). Among them, the WB abnormality is characterized by the presence of hardened and pale areas, resulting in significant economic losses for the poultry industry (Sihvo et al., 2014). In fact, the occurrence of severe WB has detrimental effects on consumer acceptance of affected fillets and, concurrently, affects their efficiency for meat further-processing (Barbut, 2019). Consequently, in the past few years, notable efforts were made to identify causative mechanisms and proper strategies to minimize the negative effects resulting from the WB emergence.

In general, the authors agree on the development of hypoxia and oxidative stress as two of the major contributors to the development of WB onset (Alnahhas et al., 2023), as demonstrated by numerous transcriptomic (Marchesi et al., 2019; Xing et al., 2021), metabolomic (Abasht et al., 2016) and proteomic studies (Bottje et al., 2021; Schmidt et al., 2023). Several studies have also evidenced dysregulations of energy metabolism (Zambonelli et al., 2016; Papah and Abasht, 2019; Lake and Abasht, 2020; Wang et al., 2023), mitochondrial dysfunction (Papah et al., 2017; Hosotani et al., 2020; Wang et al., 2023), as well as a profound alteration of the muscle fibers and extracellular matrix composition (Clark and Velleman, 2017; Velleman, 2020; Bordini et al., 2021, 2022) among the mechanisms underlying WB occurrence and progression. Nonetheless, even though detailed descriptions of the molecular mechanisms potentially involved in WB abnormality have been reported in the literature, connections among the most common molecular and physiological features characterizing its occurrence and the precise initial causes remain to be clarified.

In this scenario, the present research aimed at further characterizing broiler breast muscles affected with WB condition by providing new insights into the biological mechanisms underlying this defect and potentially identifying novel genes likely involved in its occurrence. To this purpose, raw data obtained in a previous study through RNA-sequencing technology (Mutryn et al., 2015) has been used to identify differentially expressed genes (**DEGs**) between broilers’ breast muscles affected and unaffected with WB. The most recent chicken reference genome (bGalGal1.mat.broiler.GRCg7b) was used, and differential expression analysis was carried out applying different and updated bioinformatic tools compared to those used in the previous research to advance knowledge about WB defect. In detail, revisiting RNA-seq data by using new and updated software tools, along with using the newest reference genome and taking into consideration the most recent knowledge reported in the literature concerning WB underlying causes, allowed us to get new insights into the knowledge about the complex mechanisms involved in WB occurrence. More specifically, the updated version of the cuffdiff software tool (v2.2.1) and edgeR package (v3.38.4) have been used to get a more comprehensive differential expression profile for the transcriptome of breast muscles affected by WB. Indeed, multiple bioinformatic pipelines can be used to strengthen the results and get a more extensive overview of the results, when computer resources permit (Liu et al., 2022). Therefore, for the present study, we considered using both the methods as a valid and suitable approach to further investigate the data set and use it to its full potential, and thus to deepen the knowledge about molecular mechanisms likely underlying the occurrence of WB. Moreover, functional analyses have been performed to deeply investigate molecular and biological pathways likely involved in the progression of this muscular abnormality. The results evidenced new players and likely major contributing factors involved in the occurrence and progression of WB.

## MATERIALS AND METHODS

### Samples and RNA-Seq Analysis

RNA-seq raw data obtained in a previous study (Mutryn et al., 2015) has been used to further characterize the gene expression profile of *Pectoralis major* muscle (**PM**) samples affected with WB defect. Sample collection and use for research were approved by the University of Delaware Agricultural Animal Care and Use Committee (protocol number: 44 12-15-13R). A detailed description of sample preparation, including bird housing, breast muscle sampling and classification, together with RNA extraction and library construction, was previously reported by Mutryn et al. (2015). RNA-seq data was also validated by using NanoString nCounter technology (Mutryn et al. 2015). Briefly, eleven samples of PM collected from high breast meat yield chickens (all male and sacrificed at 47 d of age) were first classified as 5 affected and 6 unaffected and then re-classified as 6 affected and 5 unaffected, following the heatmap gene cluster classification as well as unsupervised clustering of samples using metabolite profiles reported in the previous studies (Mutryn et al., 2015; Abasht et al., 2016). Bioinformatic analysis was carried out through the Biomix High-Performance Computing Cluster at the Delaware Biotechnology Institute, University of Delaware. The quality of raw RNA-seq reads was checked using FastQC v0.11.9 (Andrews, 2010), and MultiQC v1.13 (Ewels et al., 2016) was used to analyze the FastQC results. Sequence reads were trimmed using trimmomatric v.0.39 (Bolger et al., 2014), and then mapped to the newest chicken genome assembly (GRCg7b; Jan 2021) using the HISAT2 v2.2.1 aligner (Pertea et al., 2016). HISAT2 parameters were set defining strand-specific information for paired-end reads (–rna-strandness RF) and requiring only concordant alignments (–no-discordant). Mapping outputs have been converted from SAM to BAM files using SAMtools v.0.1.19 (Li et al., 2009).

### Genes Counting and Differential Expression Analyses

The DEGs between affected and unaffected samples have been identified using two different approaches: the first one regarded the usage of the newest version of cuffdiff v2.2.1 (Trapnell et al., 2012), and the second one was based on the application of edgeR package v3.38.4 (Robinson et al., 2010) in R environment (R Core Team., 2020). Considering the first approach, cuffdiff was used to both estimate gene expression abundance and identify genes differentially expressed between the two conditions (i.e., affected and unaffected chickens). To do this, the software estimated the Fragments Per Kilobase of transcript per Million mapped reads of each gene and examined the change in its expression between the affected and unaffected conditions. Genes with a false discovery rate (**FDR**) adjusted *P-value* lower than 0.05 have been considered statistically significant by cuffdiff. Also, the “cummeRbund” R package v2.38.0 has been used to manage and visualize cuffdiff outputs (Goff et al., 2023). As regards the edgeR methods, HTseq v0.11.2 (Putri et al., 2022) was used to categorize the mapped reads and obtain raw count data to be used for downstream analysis. The edgeR package allowed us to perform the normalization of the sequenced reads and the differential expression analysis. Considering the high number of genes found as statistically significant by edgeR, an FDR-adjusted *P-value* of 0.01 was considered as the significance threshold. To evaluate consistency between the two approaches, a Venn Diagram was constructed using the “VennDiagram” package v1.7.3 in the R environment.

### Functional Enrichment Analysis

Lists of significant DEGs obtained from both cuffdiff and edgeR methods were used to perform two different functional analyses. More in detail, gene lists were individually submitted to the Database for Annotation, Visualization, and Integrated Discovery (**DAVID**; version 2021) and to the ClueGO Cytoscape plugin (Bindea et al., 2009) to point out the most relevant functional terms associated with the two given DEGs lists. In particular, DAVID online tool – a popular bioinformatics resource system – has been provided with the two lists of DEGs in order to understand the biological meaning and functional grouping of the proteins coded by the considered genes. This online tool uses *kappa* statistics to measure the gene-gene functional similarity, which represents the degree of relationship between genes (Huang et al., 2007). Thus, the Functional Annotation Clustering function was considered to outline the most relevant functional terms associated with the two DEG lists. As for ClueGO, it is a user-friendly Cytoscape plug-in that allows users to analyze and visualize how functional terms are interconnected by clustering them depending on the term-term similarity, which is measured by the corrected *kappa* statistics (Bindea et al. 2009).

For both the enrichment analyses, GO terms (biological processes – BP; molecular function – MF; cellular component CC) and KEGG (Kyoto Encyclopedia of Genes and Genomes) (Kanehisa and Goto, 2000) pathways have been taken into consideration as functional categories. Also, a Benjamini-adjusted *P-value* of 0.05 was chosen as the significance threshold to identify the most significant functional terms, and *Homo sapiens* was used as the reference organism.

## RESULTS

### Reads Quality Assessment and Alignment

The number of paired raw reads per sample ranged from 32,545,784 to 57,048,229 (overall alignment from 78.41 to 83.69%). After trimming low-quality reads (Phred score < 28) and adaptor sequences, the number of mapped paired reads ranged from 30,299,181 to 51,945,676, with an overall alignment rate of paired reads mapping against the chicken reference genome ranging from 88.33 to 92.37%. MultiQC results showing mean quality scores of reads before and after trimming are reported in Supplementary Figure S1.

### Identification of Differentially Expressed Genes Using cuffdiff

The differential gene expression analysis was performed by comparing gene expression profiles of samples affected and unaffected by WB defect. Concerning the cuffdiff output, a total of 2,454 genes have been found as differentially expressed (FDR-adjusted *P-value* < 0.05; log2 fold-change > |0.4|) between the two groups. More precisely, 1,250 genes were found as upregulated in affected samples, whereas 1,204 were downregulated in the same group. A summary of the top 10 up- and downregulated genes in birds affected with WB is reported in Table 1.

**Table 1:** Top 10 up- and downregulated genes in Wooden Breast samples detected using cuffdiff approach. FDR-adjusted *P-value* < 0.05.

Top 10 upregulated			
Ensembl gene_id	Gene name	Description	log2(FC)
ENSGALG00010013765	PRLL	Prolactin like	6.569
ENSGALG00010029958	-	lncRNA	6.143
ENSGALG00010021030	CSRP3	Cysteine and glycine rich protein 3	5.944
ENSGALG00010021313	KPNA7	Laryopherin subunit alpha 7	5.768
ENSGALG00010004133	CRH	Corticotropin releasing hormone	5.127
ENSGALG00010016546	C1QTNF12	C1q and TNF related 12	4.856
ENSGALG00010013288	CA3A	Carbonic anhydrase 3A	4.437
ENSGALG00010014488	-	uncharacterized protein	4.195
ENSGALG00010012868	CTHRC1	Collagen triple helix repeat containing 1	4.179
ENSGALG00010004760	THBS2	Thrombospondin 2protein	4.120

Top 10 downregulated			

Ensembl gene_id	Gene name	Description	log2(FC)

ENSGALG00010004995	PIT54	PIT54 protein	−5.688
ENSGALG00010014087	-	lncRNA	−5.634
ENSGALG00010026526	-	lncRNA	−4.972
ENSGALG00010018616	-	uncharacterized protein	−4.789
ENSGALG00010014972	FGA	Fibrinogen alpha chain	−4.784
ENSGALG00010014956	FGG	Fibrinogen gamma chain	−4.661
ENSGALG00010005070	ALB	Albumin	−4.659
ENSGALG00010028053	ORM1	Orosomucoid 1 (ovoglycoprotein)	−4.389
ENSGALG00010016147	FABP1	Fatty acid binding protein 1	−4.377
ENSGALG00010028475	AMBP	Alpha-1-microglobulin/bikunin	−4.352

### Identification of Differentially Expressed Genes Using edgeR

A total of 2,441 DEGs were found using edgeR package in R environment by comparing affected and unaffected samples and choosing an FDR-adjusted *P-value* of 0.01 as the threshold for significance. For each DEG, log2 fold-change was greater than 0.4 (absolute value). Using an FDR of 0.05 as the significance threshold, a total of 3,967 DEGs were found. Therefore, a restricted level of significance was chosen to select genes for further analysis (i.e., functional enrichment analysis). Among the selected DEGs (FDR < 0.01), 1,378 and 1,063 genes were found respectively up- and downregulated in affected samples. A summary of the top 10 up- and downregulated genes in birds affected with WB is reported in Table 2.

**Table 2:** Top 10 up- and downregulated genes in Wooden Breast samples detected using edgeR approach. FDR-adjusted *P-value* < 0.01.

Top 10 upregulated			
Ensembl gene_ID	Gene name	Description	logFC
ENSGALG00010012474	ASB18	Ankyrin repeat and SOCS box containing 18	8.625
ENSGALG00010022690	AICDA	Activation induced cytidine deaminase	7.826
ENSGALG00010007772	CLEC3A	C-type lectin domain family 3 member A	7.639
ENSGALG00010013765	PRLL	Prolactin like	6.791
ENSGALG00010012923	MLANA	Melan-A	6.025
ENSGALG00010021313	KPNA7	Karyopherin subunit alpha 7	6.000
ENSGALG00010021030	CSRP3	Cysteine and glycine rich protein 3	5.998
ENSGALG00010018838	WFIKKN1	WAP, follistatin/kazal, immunoglobulin, kunitz and netrin domain containing 1	5.992
ENSGALG00010004296	-	uncharacterized protein	5.859
ENSGALG00010025922	-	lncRNA	5.710

Top 10 downregulated			

Ensembl gene_ID	Gene name	Description	logFC

ENSGALG00010018616	-	uncharacterized protein	−4.993
ENSGALG00010028053	ORM1	Orosomucoid 1 (ovoglycoprotein)	−4.831
ENSGALG00010028475	AMBP	Alpha-1-microglobulin/bikunin	−4.639
ENSGALG00010014956	FGG	Fibrinogen gamma chain	−4.614
ENSGALG00010026526	-	lncRNA	−4.612
ENSGALG00010005070	ALB	Albumin	−4.430
ENSGALG00010025118	-	lncRNA	−4.417
ENSGALG00010006037	METTL21EP	Methyltransferase like 21E, pseudogene	−4.318
ENSGALG00010016147	FABP1	Fatty acid binding protein 1	−4.311
ENSGALG00010014944	FGB	Fibrinogen beta chain	−3.970

### Comparison Between Cuffdiff and edgeR Methods

**Figure 1:**
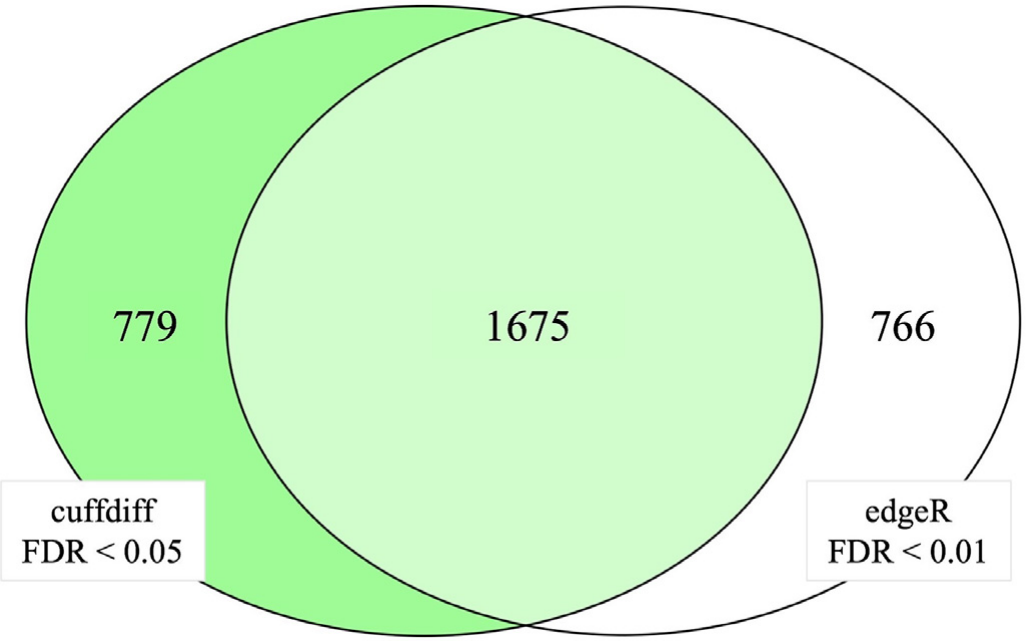
Venn diagram between cuffdiff (FDR < 0.05) and edgeR (FDR < 0.01) results for the differentially expressed genes analysis.

To evaluate consistency between cuffdiff and edgeR methods for DEG analysis, a Venn diagram was built considering gene lists obtained using the 2 different approaches (Figure 1). In particular, we compared the list of DEGs detected using cuffdiff (FDR < 0.05) to the gene list obtained using restricted parameters of significance (FDR < 0.01) for the edgeR package, since those are the groups of DEGs selected for further analysis. The Venn diagram (Figure 1) showed that even though 779 and 766 DEGs were specifically determined by cuffdiff and edgeR respectively, a total of 1675 DEGs have been identified by both methods, thus manifesting the consistency of the results. Moreover, we evaluated the consistency between results obtained considering the same FDR for both the approaches (i.e., FDR < 0.05) (Supplementary Figure S2), which further supported the consistency of the two methods by evidencing a total of 2,112 DEGs detected by both cuffdiff and edgeR.

The higher number of DEGs detected by edgeR compared to cuffdiff outputs is in line with evidence already reported in the literature (Zhang et al. 2014; Liu et al. 2022), which generally observed a higher number of DEGs detected by edgeR when compared to other software tools (e.g., Cuffdiff and DESeq2). Since this may introduce a higher number of false positives (Zhang et al. 2014), as previously mentioned, a restricted level of significance was chosen to select DEGs obtained using edgeR (FDR< 0.01) to be considered for the subsequent analysis (i.e., functional enrichment analysis).

### Functional Annotation of Differentially Expressed Genes

The lists of DEGs detected by both cuffdiff and edgeR methods were individually submitted to the DAVID online tool to perform functional enrichment analysis and thus identify over-represented pathways. The top 10 most significant functional categories of both DEGs lists identified by the DAVID tool are reported in Table 3. Interestingly, glycogen metabolic process and protein phosphorylation have been identified as significant functional terms of DEG lists obtained by using both edgeR and cuffdiff methods.

**Table 3:** The significantly enriched GO terms and KEGG pathways identified by DAVID tools considering the differentially expressed genes between affected and unaffected with Wooden Breast defect detected by the two approaches implemented in the present study: cuffdiff and edgeR.

Functional annotation of DEGs identified by cuffdiff			
Category	Term	Gene count	FDR
KEGG_PATHWAY	hsa04510:Focal adhesion	58	1.33E-08
KEGG_PATHWAY	hsa04921:Oxytocin signaling pathway	41	3.62E-05
GOTERM_CC_DIRECT	GO:0005581∼collagen trimer	26	5.55E-05
GOTERM_BP_DIRECT	GO:0005977∼glycogen metabolic process	15	3.66E-04
KEGG_PATHWAY	hsa04022:cGMP-PKG signaling pathway	38	2.21E-03
KEGG_PATHWAY	hsa05226:Gastric cancer	35	2.21E-03
KEGG_PATHWAY	hsa04151:PI3K-Akt signaling pathway	66	2.55E-03
GOTERM_BP_DIRECT	GO:0006468∼protein phosphorylation	81	2.93E-03
GOTERM_BP_DIRECT	GO:0007229∼integrin-mediated signaling pathway	27	4.73E-03
KEGG_PATHWAY	hsa04152:AMPK signaling pathway	25	9.00E-03

Functional annotation of DEGs identified by edgeR			

Category	Term	Gene count	FDR

GOTERM_CC_DIRECT	GO:0031012∼extracellular matrix	56	4.49E-07
GOTERM_BP_DIRECT	GO:0035556∼intracellular signal transduction	82	4.68E-05
GOTERM_CC_DIRECT	GO:0005576∼extracellular region	258	9.60E-05
GOTERM_BP_DIRECT	GO:0006096∼glycolytic process	17	8.87E-04
GOTERM_BP_DIRECT	GO:0005977∼glycogen metabolic process	14	1.65E-03
KEGG_PATHWAY	hsa01230:Biosynthesis of amino acids	22	3.48E-03
KEGG_PATHWAY	hsa00010:Glycolysis / Gluconeogenesis	19	1.12E-02
GOTERM_BP_DIRECT	GO:0006468∼protein phosphorylation	76	1.48E-02
GOTERM_MF_DIRECT	GO:0004674∼protein serine/threonine kinase activity	62	2.75E-02
KEGG_PATHWAY	hsa01200:Carbon metabolism	25	3.59E-02

FDR, false discovery rate.

**Figure 2:**
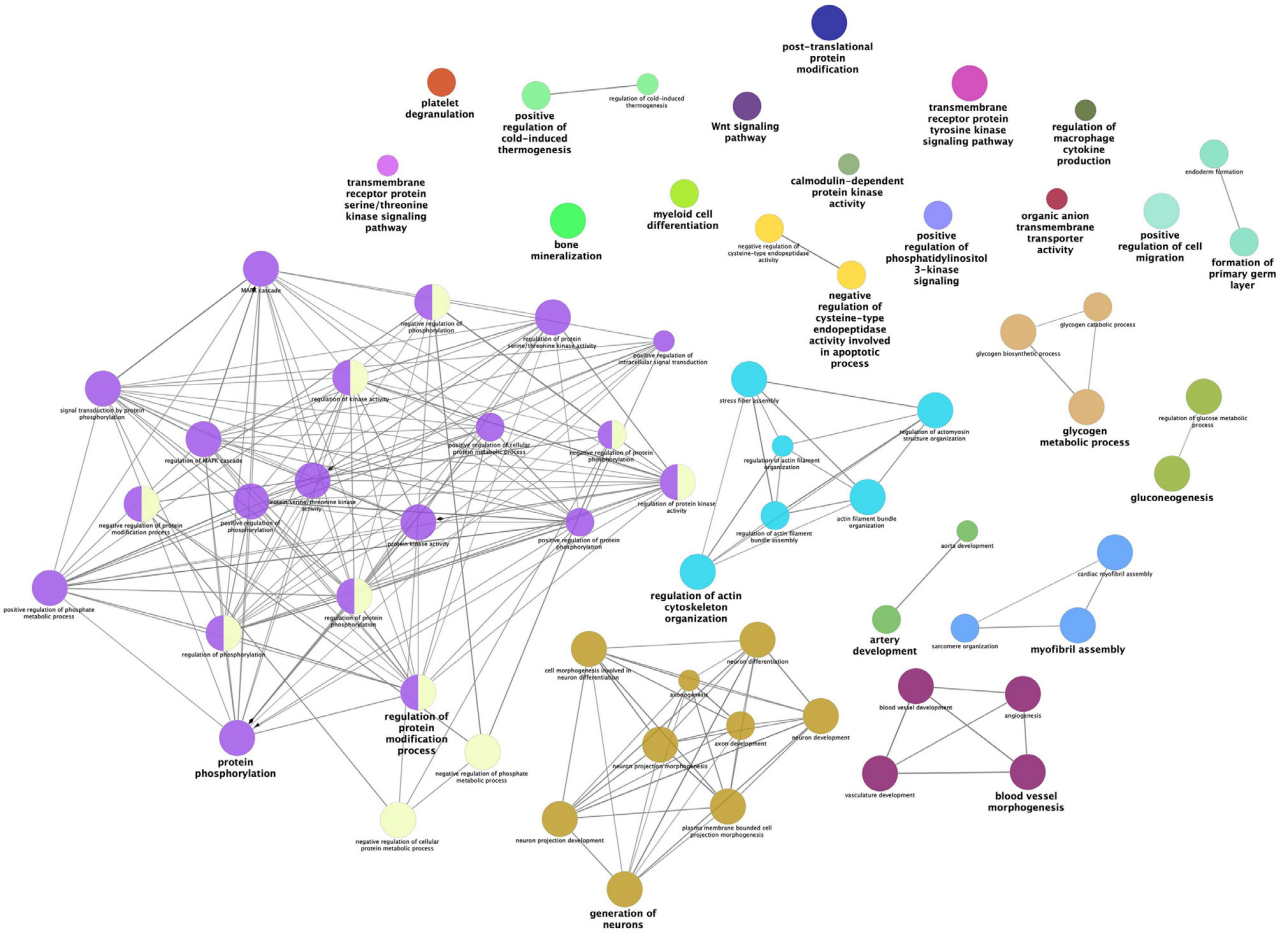
Functional network of differentially expressed genes (DEGs) identified by cuffdiff (FDR < 0.05). Terms belonging to the same functional group are colored with the same color. The size of the nodes reflects the enrichment significance of the terms and bold terms indicate the leading group term identified by the highest level of significance, using the Benjamini-Hochberg *P-value* and setting *P* < 0.05 as the significance threshold.

**Figure 3:**
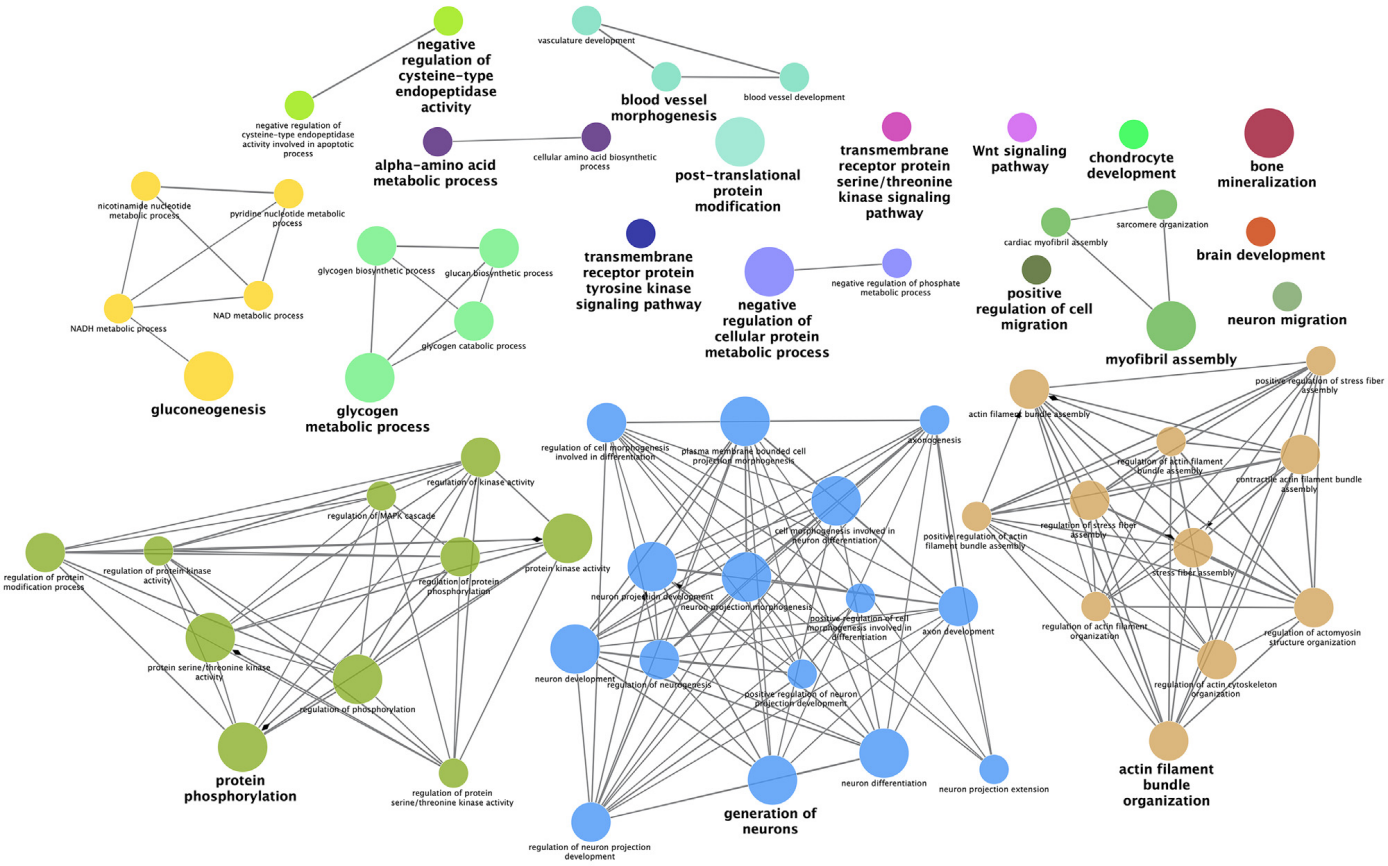
Functional network of differentially expressed genes (DEGs) identified by edgeR (FDR < 0.01). Terms belonging to the same functional group are colored with the same color. The size of the nodes reflects the enrichment significance of the terms and bold terms indicate the leading group term identified by the highest level of significance, using the Benjamini-Hochberg *P-value* and setting *P* < 0.05 as the significance threshold.

Furthermore, ClueGO functional terms analysis allowed us to deeply characterize DEGs obtained in the present study and visualize interconnections between significant functional terms (Benjamini-Hochberg adjusted *P-value* < 0.05). Particularly, ClueGO allowed us to investigate interconnections of functional terms in biological networks, by creating functional groups based on the term-term similarity calculated using *kappa* statistics. More in detail, the ClueGO plugin created annotation networks by clustering functional terms in different groups based on their similarities. Terms colored in the same way belong to the same cluster of functional terms, while terms reported in bold indicate the leading group term – according to the highest significance related to Benjamini-Hochberg – and the size of the nodes reflects the enrichment significance of the terms (Bindea et al. 2009). Figures 2 and 3 show the results obtained by analyzing DEGs obtained by cuffdiff and edgeR approaches, respectively. Glycogen metabolic process, gluconeogenesis, protein phosphorylation, myofibril assembly, blood vessel morphogenesis, and generation of neurons were the most interconnected functional terms that have been identified by ClueGO from both DEGs lists (i.e., cuffdiff and edgeR). The comprehensive results obtained from the DAVID tool and ClueGo analysis are reported in Supplementary Table S1.

## DISCUSSION

The notable efforts made trying to elucidate the underlying mechanisms of WB abnormality have led to a wide comprehension of the molecular pathways and physiological events involved in its occurrence, leading to numerous hypotheses concerning the precise initial causes of this muscular abnormality. Although a common theory has not been reported yet in the literature, most of the scientists working on this topic agree on factors likely associated with the onset of the WB defect in PMs of modern hybrids (Abasht et al., 2019a; Barbut, 2019; Petracci et al., 2019; Alnahhas et al., 2023). Indeed, hypoxia, oxidative stress, altered energy metabolism, and mitochondrial dysfunction have been considered by several authors as major contributors to the development of WB, as suggested by results obtained from omics studies (Papah et al., 2018; Brothers et al., 2019; Lake et al., 2019; Malila et al., 2021; Wang et al., 2023). Nonetheless, the accurate sequence of events leading to WB onset, as well as its precise etiology, has not been determined yet. In this scenario, the present study was intended to shed new light on mechanisms associated with the main biological and molecular alterations characterizing WB abnormality. To this purpose, we used two different approaches to detect DEGs to further investigate the data set by Mutryn et al. (2015), ultimately identifying genes that might be interesting to be considered to deepen the knowledge about molecular mechanism underlying the occurrence of WB, and that have not been reported in the literature yet.

### Muscular Hypertrophy

**Figure 4:**
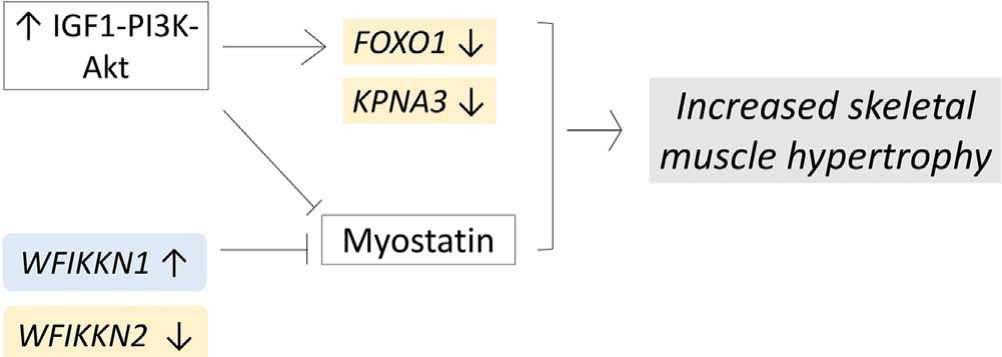
Schematic representation of proposed interaction of pathways and differentially expressed genes (DEGs) in Wooden Breast (**WB**) samples. Yellow and light-blue colors respectively indicate the lower and higher expression of DEGs related to muscular hypertrophy in WB samples. *Own design*.

As already reported in the literature and previously mentioned in this paper, selection for muscular hypertrophy seems to have had a relevant role in the occurrence of breast muscle defects affecting fast-growing broiler genotypes (Soglia et al., 2021). In this context, the *WFIKKN1* chicken gene, identified as one of the top 10 upregulated DEGs in samples affected by WB and detected by edgeR, may be considered of particular interest. Indeed, this gene encodes for an extracellular multidomain protein (also called GASP) that binds semi-latent myostatin (a negative regulator of skeletal muscle growth) and inhibits its activity (Szláma et al., 2013), thus promoting skeletal muscle growth (Lee and Lee, 2013; Wang et al., 2015). In this regard, the overexpression of *WFIKKN1* in WB samples may be related to the proneness of hypertrophic growth characterizing the PMs in fast-growing hybrids (Figure 4; *own design*). On the other hand, our findings also evidenced a downregulation in WB samples of *WFIKKN2*, the gene encoding for the homologous form of WFIKKN1 protein. This result may be explained considering that this form of WFIKKN protein mainly binds the mature form of myostatin, and, most importantly, that only *WFIKKN1* directly interacts with the myostatin precursor (Szláma et al., 2016) (i.e., pro-myostatin) (Anderson et al., 2008), thus suggesting a different activity of the two forms of myostatin inhibitors. Moreover, if compared to *WFIKKN1*, a lower efficiency of *WFIKKN2* in suppressing myostatin activity has been reported in the literature (Szláma et al., 2013), therefore indicating a greater potential of the former in promoting muscular hypertrophy.

Concerning myostatin activity and muscle hypertrophy, Wnt and PI3K-Akt signaling pathways are considered critical modulators. More in detail, IGF1-PI3K-Akt pathways can have a pro-hypertrophic activity by inhibiting myostatin (Glass, 2010; Retamales et al., 2015), thus resulting in an increased muscle mass. Moreover, the PI3K-Akt signaling pathway – found as one of the significant functional categories detected by DAVID tool in the present study – regulates cell size (e.g., skeletal muscle cells) via FOXO proteins, that, among different functions, play critical roles in the regulation of cellular growth (Li et al., 2019). In particular, the FOXO1 protein, encoded by the homonymous genes, is known to regulate skeletal muscle development by exerting negative regulations on muscle cell differentiation (Xu et al., 2016). Recent studies aimed at investigating genetic factors associated with growth traits in modern broilers by using a genome-wide association approach evidenced that the *KPNA3*-*FOXO1* region is potentially involved in breast and leg muscle growth (Xie et al., 2012). Interestingly, both *FOXO1* and *KPNA3* genes were downregulated in WB samples in the present study, thus supporting their potential involvement in affecting chicken growth traits and corroborating the association between muscle hypertrophy and WB occurrence (Figure 4; *own design*). Moreover, FOXO1 is involved in the regulation of energy metabolism (Bastie et al., 2005; Peeters et al., 2011), and dysregulation of *FOXO1* gene expression has been associated with increased lipid accumulation and gluconeogenesis suppression (Bastie et al., 2005; Peeters et al., 2011). In light of the above, the present results might suggest that genetic selection intended to increase muscular hypertrophy in meat-type chickens may have indirectly led to negative regulations of genes involved in the regulation of energy metabolism (e.g., *FOXO1*). Most interestingly, *FOXO1* is specifically regulated by the peroxisome proliferator-activated receptor co-activator 1 (**PGC1s**) family, which, in the present research, has been found downregulated in WB samples, and plays a critical role in promoting mitochondrial biogenesis (Ventura-Clapier et al., 2008).

Curiously, both enrichment analyses found “generation of neurons” as a leading term of a functional network of DEGs. Even if further analysis will be necessary to investigate the relationship between genes related to neuron development and migration and WB, it is worth noting that several genes enriched in this functional network encode for Wnt proteins (e.g., *WNT1, WNT11, WNT16, WNT5B, and WNT9A*), along with genes encoding isoforms of the catalytic subunit of phosphoinositide 3-kinase (**PI3K**; *PIK3CB,* and *PIK3CD*). It might be then speculated that genes enriching the generation of neurons’ functional network may be related to signaling pathways such as Wnt and PI3K-Akt signaling pathways, that, in turn, are known to be modulators of cellular growth and muscle hypertrophy (von Maltzahn et al., 2012).

### Mitochondrial Dysfunction and Energy Metabolism

**Figure 5:**
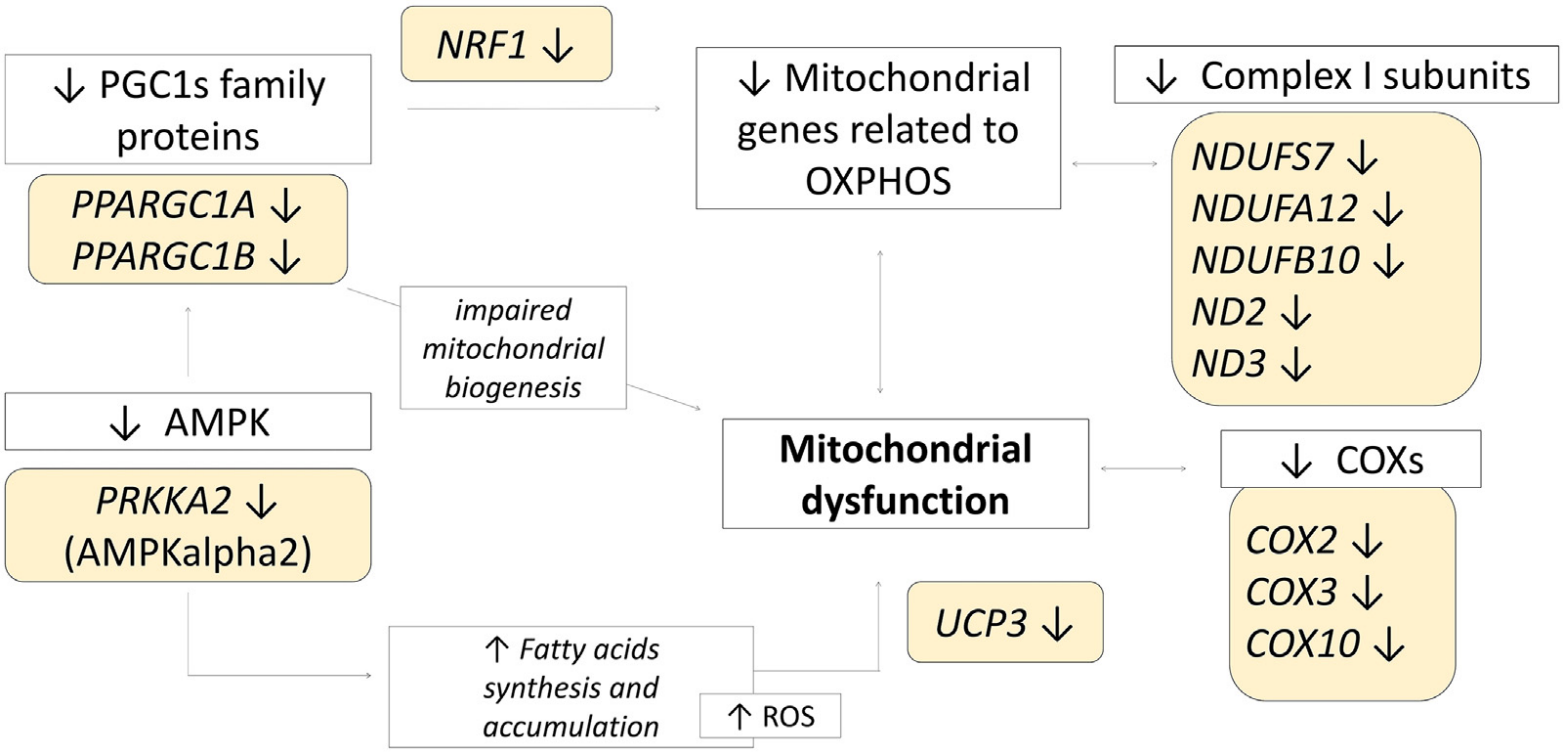
Schematic representation of proposed interaction of pathways and differentially expressed genes (**DEGs**) in Wooden Breast (**WB**) samples. Yellow color and arrows indicate the lower expression of DEGs related to mitochondrial dysfunction and fatty acid metabolism in WB samples. *Own design*.

The PGC1s family consists of transcriptional coactivators playing central roles in molecular functions that integrate mitochondrial functionality and energy production at the cellular level (Scarpulla, 2010). More specifically, PGC-1α and PGC-1β, two factors belonging to the PGC1s family, are reported to be intensive promoters of mitochondrial biogenesis (Ventura-Clapier et al., 2008; Scarpulla, 2010; Cheng et al., 2018) (Figure 5). Indeed, the expression level of their respective coding genes, *PPARGC1A* and *PPARGC1B*, plays critical roles in coordinating gene expression of mitochondrial key components, such as the oxidative phosphorylation complexes (Scarpulla, 2010; Cheng et al., 2018). PGC1s activity is, in turn, regulated by the AMP-activated protein kinase (**AMPK**) (Peeters et al., 2011; Fuentes et al., 2013; Herzig and Shaw, 2018). Interestingly, Buemann and Uvnäs-Moberg (2020) hypothesized that AMPK activity could be induced by oxytocin. The authors also evidenced that oxytocin may have a role in protecting cells against inflammatory stress and degeneration. This seems to be in line with our results, which have pointed out the “Oxytocin signaling pathway” as one of the functional categories identified by the DAVID analysis from the results generated by cuffdiff. Supporting this evidence, genes encoding the AMPK and calmodulin-dependent protein kinase (**CAMK**) pathways were enriched in this functional category (e.g., *PRKAA2, CAMK4, CAMK1D, CAMK2A*). It is worth mentioning this AMPK pathway, enhanced by the CAMK pathway, has been shown both to promote the autophagy of mitochondria when damaged and to promote mitochondria biogenesis (Herzig and Shaw, 2018; Buemann and Uvnäs-Moberg, 2020).

In this regard, several studies report that AMPK pathway stimulates mitochondrial biogenesis via PGC-1α induction (Peeters et al., 2011; Herzig and Shaw, 2018; Kjøbsted et al., 2018). More in detail, PGC1s modulate the expression level of genes encoding for electron transport chain (**ETC**) subunits and mtDNA genes by inducing the *NRF1* gene transcription (Wu et al., 1999; Ventura-Clapier et al., 2008; Scarpulla, 2010). This gene, together with the *NRF2*, is involved in the biogenesis of the respiratory chain complexes, such as the cytochrome c (Scarpulla, 2010).

In the present study, *PPARGC1A* was found downregulated in WB using the cuffdiff approach, and *PPARGC1B* has been found lower expressed in affected samples using both edgeR and cuffdiff methods. In line with this outcome, *NRF1* and *PRKAA2* (which is one of the genes encoding for AMPK) were found downregulated in WB as well. Because of their role in regulating mitochondrial biogenesis, the downregulation of genes coding for PGC-1α and PGC-1β factors, as well as their downstream player (i.e., *NRF1*) and upstream regulator (i.e., *PRKAA2*), may be considered likely related to the mitochondrial dysfunction hypothesized in WB muscles (Papah et al., 2017; Hosotani et al., 2020; Hasegawa et al., 2022; Wang et al., 2023). In line with these results, the present research evidenced other genes involved in the pathways of oxidative phosphorylation as lower expressed in WB, such as *ND2* and *ND3* (Mimaki et al., 2012), as well as genes coding for the mitochondrial cytochrome c oxidase (e.g., *COX1* and *COX3*) (Timón-Gómez et al., 2018). Therefore, the downregulation of these genes might be involved in the dysregulation of the mitochondrial functions and ROS production already described in WB-affected chickens.

Furthermore, PGC-1α is known to be involved in energy metabolism by promoting the expression of genes involved in the tricarboxylic acid cycle and the mitochondrial fatty acid oxidation pathway (Cheng et al., 2018). Most interestingly, several authors highlighted the relevant role of PGC1s in regulating lipid metabolism. In particular, the downregulation of its coding genes has been associated with increased fatty acid uptake and lipid accumulation at the muscular level (Espinoza et al., 2010; Supruniuk et al., 2017), consistent with the lipid toxicity hypothesized in WB muscles and likely underlying the occurrence of the defect (Lake and Abasht, 2020; Wang et al., 2023). In addition, PGC1s play relevant roles in inducing the mRNA expression of the uncoupling proteins 3 (**UCP3**), which protects mitochondria from lipotoxicity by reducing ROS production from fatty acids at the mitochondrial level (Hoeks et al., 2006). Indeed, it has been reported that a reduced *UCP3* gene expression level is associated with mitochondrial damage induced by lipid accumulation, and results in increased ROS production at the mitochondrial level (Hoeks et al., 2006; Schrauwen et al., 2006). Considering the above, an alteration of mitochondrial mechanisms having the purpose of protecting cells from ROS accumulation toxicity – especially related to fatty acid peroxide – may be hypothesized.

It is also worth mentioning that the reduced activity of PGC1s has been associated with insulin resistance and type 2 diabetes in humans (Soyal et al., 2006; Handschin et al., 2007; Peeters et al., 2011). This evidence further supports the hypothesis concerning the similarities between the mechanisms underlying WB condition in chickens and type 2 diabetes in humans (Lake and Abasht, 2020). Overall, the lower expression of PGC1s coding genes in WB samples detected in the present study may be considered in line with evidence already reported in the literature pointing out an alteration of fatty acid metabolism and mitochondrial functionality of PMs affected by WB (Figure 5; *own design*).

### Altered Carbohydrate Metabolism and Hexosamine Pathway

**Figure 6:**
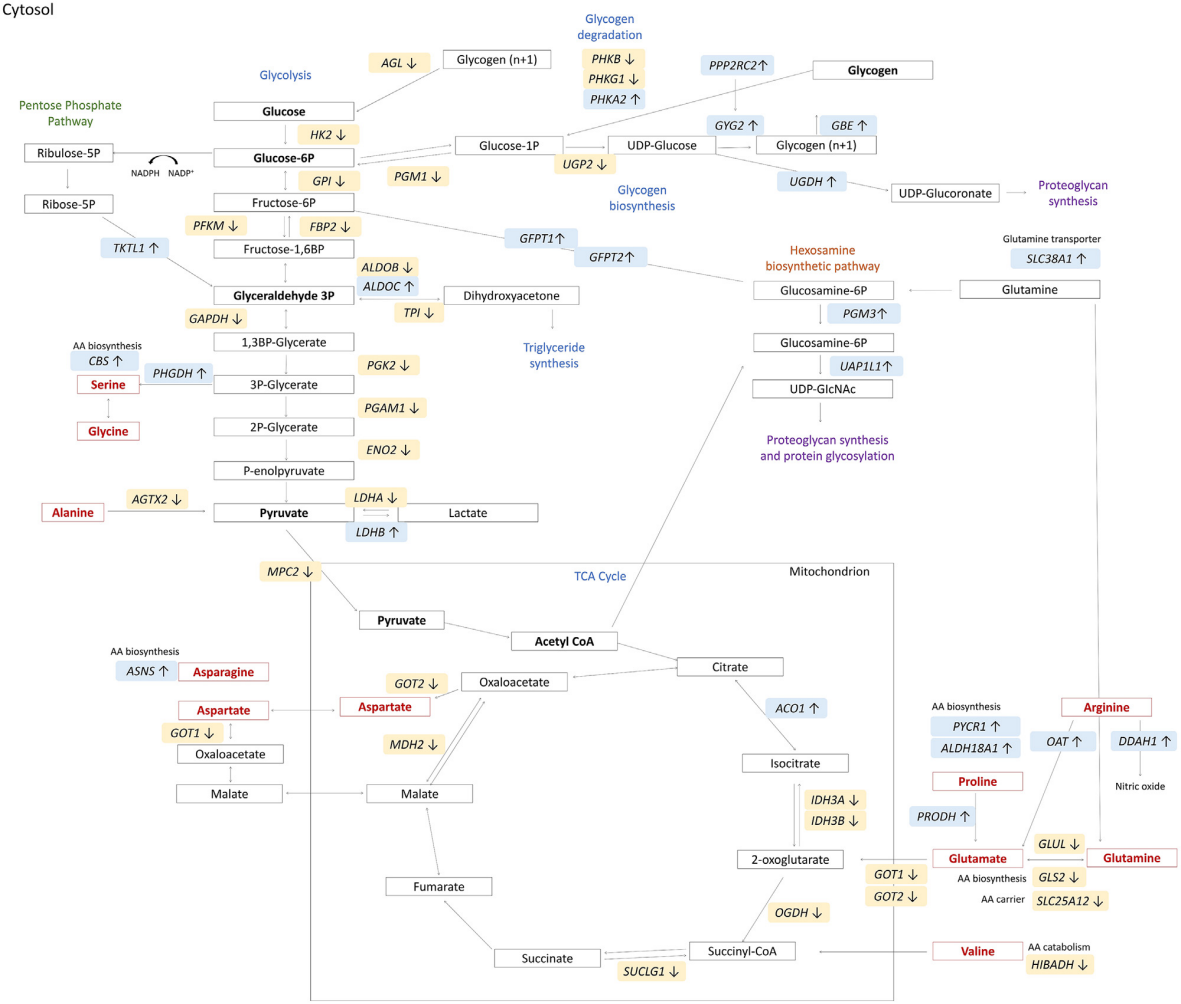
Schematic representation of differentially expressed genes (**DEG**) related to carbohydrate metabolism (in blue), hexosamine biosynthetic pathway (HBP) (in orange), and proteoglycan synthesis (in purple). Colors and arrows indicate higher (light blue; ↑) and lower (yellow; ↓) expression of DEGs. Amino acids related to carbohydrate metabolism and HBP are reported in red. *Own design*.

As regards the altered glucose metabolism characterizing fast-growing broilers affected with WB, differential expression and functional analyses highlighted that several genes involved in carbohydrate metabolism are downregulated in WB samples. Indeed, functional analyses performed using DAVID tool and ClueGO evidenced that DEGs detected both by cuffdiff and edgeR methods enriched glycogen metabolic process, glycolysis, and gluconeogenesis. Several genes related to glycolysis and gluconeogenesis pathways have been found downregulated in WB, as shown in Figure 6 (*own design*). These results are in line with previous studies reporting a reduced carbohydrate metabolism in broilers affected with WB (Abasht et al., 2016; Papah et al., 2018). Additionally, in agreement with results obtained by Papah et al. (2018) and Zambonelli et al. (2016), a potential shift of glucose usage from glycolysis to the hexosamine biosynthetic pathway (**HBP**) could be hypothesized. In fact, affected samples were characterized by an upregulation of genes considered as markers to identify the HBP status, *GFPT1* and *GFPT2* (Coomer and Essop, 2014; Kroef et al., 2022) and that encode for the fructose-6-phosphate amidotransferase (**GFPT**), the rate-limiting enzyme of the *de novo* synthesis hexosamine (Paneque et al., 2023). Interestingly, the end-product of HBP is uridine diphosphate-N-acetyl glucosamine (**UDP-GlcNAc**), a key metabolite essential for post-translational protein modification (e.g., O-GlcNAcylation) (Paneque et al., 2023), and for proteoglycans and glycosaminoglycans synthesis (Buse, 2006). Another gene found as upregulated in affected samples is *UAP1L1*. It directly interacts with the O-GlcNAc transferase enzyme and promotes O-GlcNAcylation (Lai et al., 2018), thus supporting the hypothesis of an upregulation of O-GlcNAcylation in samples affected with WB. In line with the above, other authors have suggested that HBP may be considered a critical pathway driving the occurrence and progression of WB defect in fast-growing chickens (Papah et al., 2018; Lake and Abasht, 2020; Soglia et al., 2021), for example by increasing the proteoglycans production (Velleman and Clark, 2015; Clark and Velleman, 2017). With regards to the glycosaminoglycans synthesis, *UGDH* was found upregulated in affected samples. This gene encodes for the homonymous enzyme catalyzing the UDP-glucuronate, which is an essential precursor for new extracellular matrix glycosaminoglycans (Zimmer et al., 2021).

As a new piece of information, our results may suggest a shift toward the HBP not only from the glycolysis pathway but also from glutamate metabolism. Indeed, the present results evidenced a lower expression of genes involved in glutamate biosynthesis from glutamine (e.g., *GLS2* and *SLC25A12*), together with the upregulation of genes involved in glucosamine synthesis and plasma membrane glutamine transporters (Figure 6; *own design*). Besides, genes involved in glutamate catabolism, such as *GOT1* and *GOT2*, were found downregulated in WB samples. Taken together, the present results seem to support the hypothesis of an increased HBP at the expense of the glutamate biosynthesis pathway.

## CONCLUSIONS

This study has potential limitations related to the lack of targeted validation of the discussed DEGs through a different gene expression analysis, and therefore additional investigations are required to verify the assumption reported in the present research study. Nevertheless, results achieved in the present study could be considered a starting point for future research and considerations.

Overall, the results obtained in the present study showed a downregulation of genes coding for mitochondrial biogenesis, cytochrome c subunits, and other players of the ETC. These results are in line with those hypotheses reported in the literature that pointed out mitochondrial dysfunction as one of the potential causes at the basis of WB occurrence in fast-growing broilers. Most interestingly, the *PPARGC1A* and *PPARGC1B* genes not only are involved in mitochondrial biogenesis but also play pivotal roles in maintaining lipid and glucose homeostasis. Together with these pieces of evidence, the present outcomes pointed out genes likely related to the hypertrophic growth characterizing modern broilers (e.g., *WFIKKN1*), and suggested a shift toward the HBP from the glycolysis pathway as well as from the glutamate metabolism, other than further highlighting the altered carbohydrate metabolism characterizing WB muscles. Altogether, our results corroborate the evidence concerning the impaired glucose metabolism and mitochondrial dysfunction in WB muscles and identify genes potentially involved in those mechanisms. Since the PGC1s family plays pivotal roles in maintaining both glucose homeostasis and mitochondrial biogenesis and function, its involvement in the molecular alterations characterizing WB muscles might be hypothesized. As far as we know, this is the first study identifying a direct association between the differential expression of PGC1s coding genes and the WB defect. Additional investigations are needed to verify the actual and precise roles of the genes identified as possible players in the molecular and biological pathways discussed in the present study.

## DISCLOSURES

The authors declare no conflicts of interest.

## Appendix Supplementary materials

Image, application 1

Image, application 2

## Footnotes

Supplementary material associated with this article can be found in the online version at doi:10.1016/j.psj.2024.103902.
