# Supplementary material for: RNA-sequencing revisited data shed new light on wooden breast myopathy
Source: Poult Sci. 2024 May 25;103(8):103902. doi: 10.1016/j.psj.2024.103902 (PMC11246058; doi:10.1016/j.psj.2024.103902)

**Supplementary Figures**

**Supplementary Figure S1.** MultiQC output showing Phred Quality Score of reads before (A) and after (B) trimming.

**
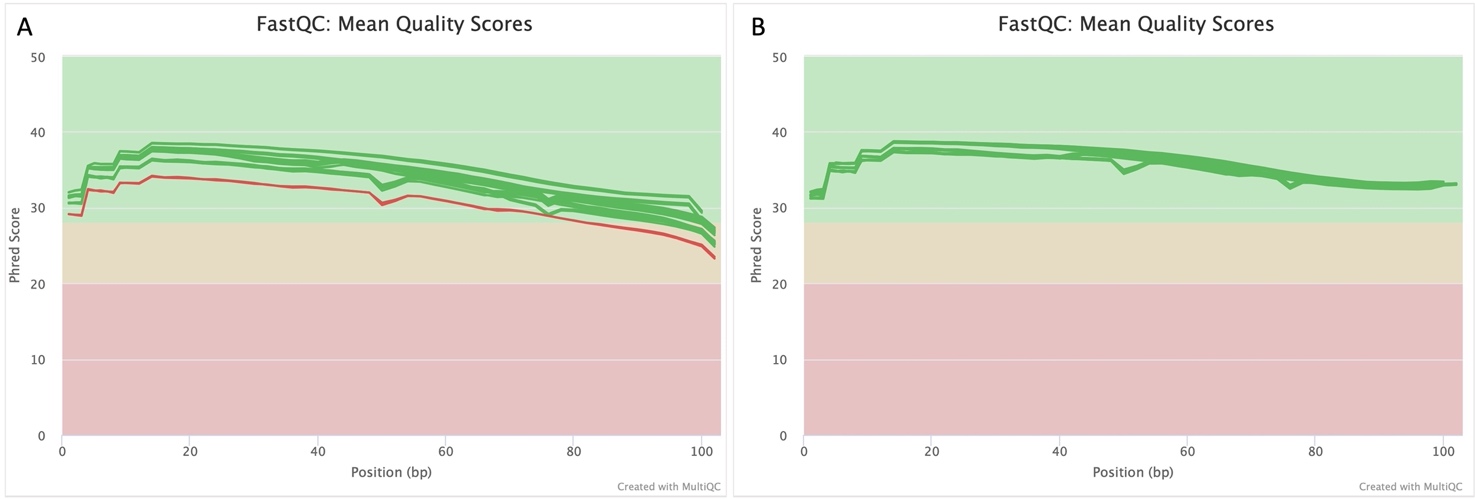
**

**Supplementary Figure S2.** Venn Diagram between cuffdiff (FDR < 0.05) and edgeR (FDR < 0.05) results for the Differentially Expressed Genes (DEG) analysis.


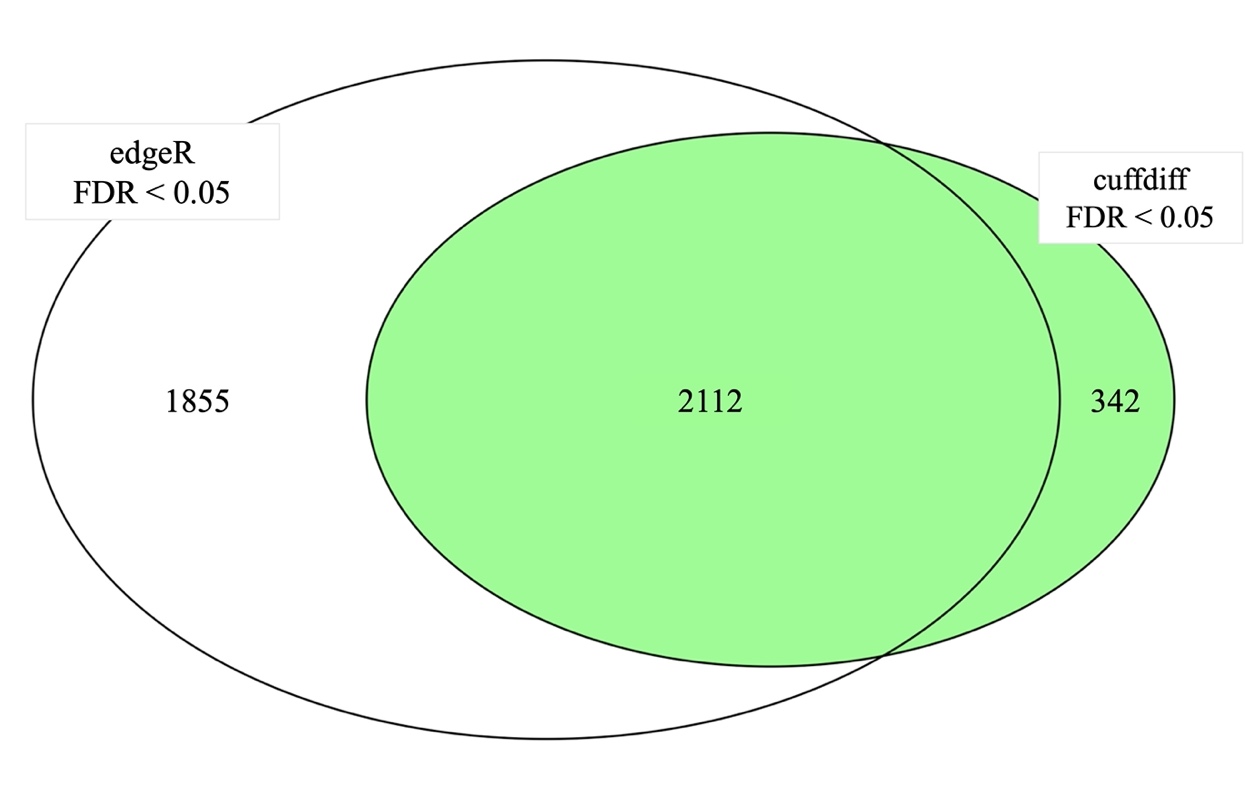

Supplement: Supplementary file 1 [file mmc1.docx]
